# Effects of Ultrasound Assisted Extraction in Conjugation with Aid of Actinidin on the Molecular and Physicochemical Properties of Bovine Hide Gelatin

**DOI:** 10.3390/molecules23040730

**Published:** 2018-03-22

**Authors:** Tanbir Ahmad, Amin Ismail, Siti A. Ahmad, Khalilah A. Khalil, Teik K. Leo, Elmutaz A. Awad, Jurhamid C. Imlan, Awis Q. Sazili

**Affiliations:** 1 Department of Animal Science, Faculty of Agriculture, Universiti Putra Malaysia, Serdang 43400, Selangor, Malaysia; tanbirvet05@rediffmail.com (T.A.); leoteikee@gmail.com (T.K.L.); 2 ICAR-Central Institute of Post-Harvest Engineering and Technology, Ludhiana, Punjab 141004, India; 3 Faculty of Medicine and Health Sciences, Universiti Putra Malaysia, Serdang 43400, Selangor, Malaysia; aminis@upm.edu.my; 4 Halal Products Research Institute, Putra Infoport, Universiti Putra Malaysia, Serdang, Selangor 43400, Malaysia; 5 Faculty of Biotechnology and Biomolecular Sciences, Universiti Putra Malaysia, Serdang, Selangor 43400, Malaysia; aqlima@upm.edu.my; 6 Faculty of Applied Sciences, Universiti Teknologi MARA, Shah Alam 40450, Selangor, Malaysia; khalilahabdkhalil@gmail.com; 7 Laboratory of Sustainable Animal Production and Biodiversity, Institute of Tropical Agriculture and Food Security, Universiti Putra Malaysia, Serdang, Selangor 43400, Malaysia; motazata83@gmail.com (E.A.A.); jurhamidimlan@yahoo.com.ph (J.C.I.); 8 Department of Poultry Production, University of Khartoum, Khartoum 13314, Sudan; 9 Department of Animal Science, College of Agriculture, University of Southern Mindanao, Kabacan 9407, North Cotabato, Philippines

**Keywords:** ultrasound assisted extraction, gelatin, actinidin, bovine hide, physicochemical properties, gel strength

## Abstract

Actinidin was used to pretreat the bovine hide and ultrasonic wave (53 kHz and 500 W) was used for the time durations of 2, 4 and 6 h at 60 °C to extract gelatin samples (UA2, UA4 and UA6, respectively). Control (UAC) gelatin was extracted using ultrasound for 6 h at 60 °C without enzyme pretreatment. There was significant (*p* < 0.05) increase in gelatin yield as the time duration of ultrasound treatment increased with UA6 giving the highest yield of 19.65%. Gel strength and viscosity of UAC and UA6 extracted gelatin samples were 627.53 and 502.16 g and 16.33 and 15.60 mPa.s, respectively. Longer duration of ultrasound treatment increased amino acids content of the extracted gelatin and UAC exhibited the highest content of amino acids. Progressive degradation of polypeptide chains was observed in the protein pattern of the extracted gelatin as the time duration of ultrasound extraction increased. Fourier transform infrared (FTIR) spectroscopy depicted loss of molecular order and degradation in UA6. Scanning electron microscopy (SEM) revealed protein aggregation and network formation in the gelatin samples with increasing time of ultrasound treatment. The study indicated that ultrasound assisted gelatin extraction using actinidin exhibited high yield with good quality gelatin.

## 1. Introduction

Gelatin is a high molecular weight biopolymer obtained from collagen by thermal hydrolysis causing its denaturation. Being a versatile biomaterial, it is extensively used in preparing various food products, medicines, cosmetic items and in photography because of its film-forming capability, water binding ability and emulsifying and foaming properties [1,2].

Insoluble collagen is required to be converted into soluble form by pretreatment with either acid or alkali resulting in the loss of the triple-helical arrangement of native collagen chains which is swollen but still insoluble [3]. Finally, conversion into gelatin takes place during extraction process due to the cleavage of hydrogen and covalent bonds by heat leading to helix-to-coil transition [4]. Cleavage of covalent and non-covalent bonds in sufficient numbers releases free α chains and oligomers [5]. Additionally, few amide bonds present in the original collagen triple chains are broken down by hydrolysis [6]. Consequently, the recovered gelatin has lower molecular weight polypeptide chains compared to native collagen chain and the extracted gelatin represents a mixture of polypeptide chains having molecular weight ranging from 16 to 150 kDa [7].

There is lack of published research on ultrasound assisted extraction (UAE) of bioactive materials from animal sources [8,9,10]. UAE can increase extraction efficiency and extraction rate particularly for aqueous extraction and lower processing temperatures can be applied for enhanced extraction of heat sensitive bioactive food components at lower processing temperatures [11]. Its promising effect in food science has attracted attention of food industry [12]. High power ultrasound (power >1 W cm^−2^ and frequencies between 20 and 500 kHz) can be applied to aid the extraction process of different food components such as herbal, oil, protein and polysaccharides including bioactive compounds such asantioxidants from various animal and plant materials [11]. Ultrasonic irradiation increased the yield of collagen from bovine tendon and significantly shortened the extraction time in comparison to the traditional pepsin aided extraction process [13]. The extraction yield of collagen increased with the ultrasonic treatment [14]. Good quality gelatin with high yield (30.94–46.67%) was obtained from bighead carp scales by using ultrasound bath and the presence of α-and β-chains were observed in the resulting gelatin [15].

Collagen cross-links bonds are resistant to thermal and acid hydrolysis [16] resulting in a low gelatin yield [17]. Previously, some proteases capable of breaking the collagen cross-links have been used to increase the extractability of gelatin [17]. Pepsin and proctase (isolated from *Aspergillus niger*) were used to extract the gelatin from bovine hide but the gelatin yield, its gel strengths and viscosities were low [18]. Crude proteolytic enzyme from papaya latex and commercial papain were used to extract gelatin from the raw hide and higher yield was obtained but the gel strength was relatively low and complete degradation of α and β chains in the recovered gelatins were observed in both types of samples [19]. Papain was used to extract gelatin from rawhide splits but the obtained gelatin showed low gel strength and viscosity [20]. Although better gelatin yield was achieved, the functional qualities of the obtained gelatin were lowered. Gelatin with high molecular weight polymers (less degraded peptides) are reported to be better in functional properties [21,22,23,24]. Therefore, novel enzymes capable of cleaving long chains of gelatin only at few sites should be explored so that a long chain gelatin of high quality can be produced [25]. Actinidin protease was most specifically effective at hydrolysing meat myofibril proteins out of papain, bromelain, actinidin and zingibain [26]. Earlier study from this laboratory (unpublished results) showed encouraging result in term of gelatin yield and quality when bovine hide was pretreated with actinidin at level of 20 unit of enzyme per gram of hide. Therefore, actinidin has been included in this study. There is no published research work on the ultrasound assisted extraction of gelatin as well as on the ultrasound–enzyme assisted extraction of gelatin from bovine hide. Hence, the objectives of this study were to extract gelatin using ultrasound in conjugation with enzyme actinidin pretreatment and investigating their effects on the quality characteristics of the recovered gelatin.

## 2. Results and Discussion

### 2.1. Gelatin Yield

The effects of ultrasound assisted extraction in conjugation with actinidin on gelatin yield are shown in Table 1. The gelatin yield was significantly (*p* < 0.05) increased with increasing the duration of ultrasound treatment. The result was in accord with those of Arnesen and Gildberg [27] and Tu et al. [15] who reported that higher yield of gelatin was obtained with longer extraction time from Atlantic salmon skin and bighead carp scales, respectively. More energy was provided by increasing time to destroy the stabilizing bonds present in the collagen structures and peptide bonds of α-chains resulting in helix-to-coil transformation [28]. At higher temperature, conversion of collagen to gelatin is brought about by destruction of the stabilizing hydrogen bonds of collagen resulting in the transformation of helix-to-coil structure [29]. In addition, few peptides bonds are also broken down [30]. 

The higher yield of gelatin with increasing duration could also be due to cavitation and mechanical effect of ultrasound [15]. Acoustic cavitation is mainly responsible for the increased extraction obtained from UAE [31] as it releases more energy to wash out the gelatin from the hide sample. Besides, ultrasound increases the contact surface area between sample matrix and solvent by producing mechanical effect and thus enabling greater penetration of liquid medium into the solid phase for extraction [32]. Thus, a greater penetration of solvent into sample matrix and improved mass transfer was facilitated by the acoustic cavitation and mechanical effects of ultrasound [33]. Li et al. [13] observed enhanced collagen extraction with the use of ultrasound due to cavitation which opened the collagen fibrils and improved the dispersal of enzyme aggregates and this assisted in carrying molecules of pepsin in the close vicinity of collagen chains affecting the hydrolysis.

In the present study, UA6 had significantly (*p* < 0.05) higher gelatin yield compared to UAC (19.65% vs. 18.72%). This result corroborated the previous findings where higher gelatin yield was obtained with the proteolytic enzymes pretreatment [34,35,36]. Balti et al. [34] reported extraction yield increased from 2.21% to 7.84% on wet weight basis from skin of cuttle fish (*Sepia officinalis*) when smooth hound crude acid protease at level 15 units/g was used. Bougatef et al. [36] obtained 54.61% and 15.22% gelatin from skin of smooth hound in the presence and absence of smooth hound crude acid protease (SHCAP) enzyme when citric acid was used as pretreatment agent. In addition, Lassoued et al. [35] obtained higher gelatin yield from thornback ray (*Raja clavata*) skin with pepsin pretreatment. Nalinanon et al. [17] also reported markedly higher gelatin yield when proteases were added to extract the gelatin compared to the gelatin yield without enzyme.

### 2.2. Colour

Colour coordinates a* and b* of UA2 sample were significantly (*p* < 0.05) lower than the rest of the samples (Table 2). The highest lightness (L* value) for UA2 was consistent with the finding of Sinthusamran et al. [37] who reported highest L* (lightness) for gelatin extracted for short time. The higher yellowness (b* value) for UA4, UA6 and UAC samples might be due to non-enzymatic browning reaction [37].

### 2.3. pH

There was a significant (*p* < 0.05) increase in pH as the extraction time increased and the highest pH (3.03) was recorded for UA6 (Table 1). Mohtar et al. [38] reported that the pH of bovine gelatin was 5.48. The HCl used for pretreatment of hide could be a possible explanation for the lower pH in our current study. The relationship between the pH of gelatin and processing method used to extract gelatin has not been established yet [39].

### 2.4. Amino Acid Composition of Gelatin

Gelatin properties are greatly determined by the amino acid composition and molecular weight distribution [40]. The most abundant amino acid in gelatin is glycine [41]. Repeating chains of Gly-X-Y, where X and Y usually denote proline and hydroxyproline, respectively, are present in triple peptides which make up to 50–60% of α-chains [7]. A higher content of proline and hydroxyproline (imino acid) amino acids, particularly hydroxyproline, are found in the gelatins extracted from warm-blooded animal tissues [42].

There is a dearth of published research on the effects of duration of ultrasound treatment on the amino acid content. Improved hydrophobic amino acids content of rice dreg protein (RDP) extracted from rice dreg flour (RDF) using ultrasound treatment was obtained [43]. Micro fractures, molecule unfolding and protein structure changes occurred due to high-intensity shock waves, microjets, shear forces and turbulence produced as a result of cavitation effect [44] leading to increased amino acid content [43]. In present study, glycine, proline and hydroxyproline contents for UAC, UA2, UA4 and UA6 were25.54%, 11.39% and 17.00%;16.86%, 8.33% and 10.77%;18.95%, 9.26% and 12.64%; and 20.60%, 9.78% and 13.65%, respectively (Table 3). The amino acids content increased with the increase in time duration of ultrasonic treatment and UAC exhibited the highest content of amino acids.

Ox skin and calf skin contained 27.6%, 16.5% and 13.4%, and 26.9%, 14.0% and 14.6% glycine (Gly), proline (Pro) and hydroxyproline (Hyp), respectively [45]. Furthermore, Lassoued et al. [35] and Balti et al. [34] reported the glycine, proline and hydroxyproline content of food grade halal bovine gelatin as 34.48%, 13.39% and 9.54%, and 34.1%, 12.3% and 9.6% of the total amino acids, respectively. Our amino acid results are expressed in terms of percentage of sample weight (mg of amino acid per 100 mg of sample). The observed variations in the amino acid contents might be also due to differences in manufacturing processes of gelatin [46].

The amino acid (Pro + Hyp) content of UAC, UA2, UA4, UA6 and were 28.39%, 19.10%, 21.90%, and 23.43%, respectively. The imino acid content in bovine gelatin ranged between 21.90% [34,35] and 23.3% [47]. Hyp content (for UA2, UA4, UA6 and UAC were 10.77%, 12.64%, 13.65% and 17.00%, respectively) were higher than those (9.6% and 9.54%, respectively) previously reported [34,35] in halal bovine gelatin. The stability of the triple helical structure of the gelatins gel is directly dependent on the quantity of Pro and Hyp (imino acids) as nucleation zones are formed in Pro + Hyp rich areas [48]. Additionally, stability to the triple-stranded collagen helix is believed to be provided by Hyp through its ability to form hydrogen bond through its hydroxyl group [48,49]. The high imino acid content as obtained for different samples in this study was reflected in the high gel strength of the UG samples.

All data areexpressed in the unit of g/100 g of gelatin. Measurements were performed in triplicate and data correspond to mean values. Standard deviations were in all cases lower than 2%.

### 2.5. SDS-PAGE Analysis of Gelatin

**Figure 1:**
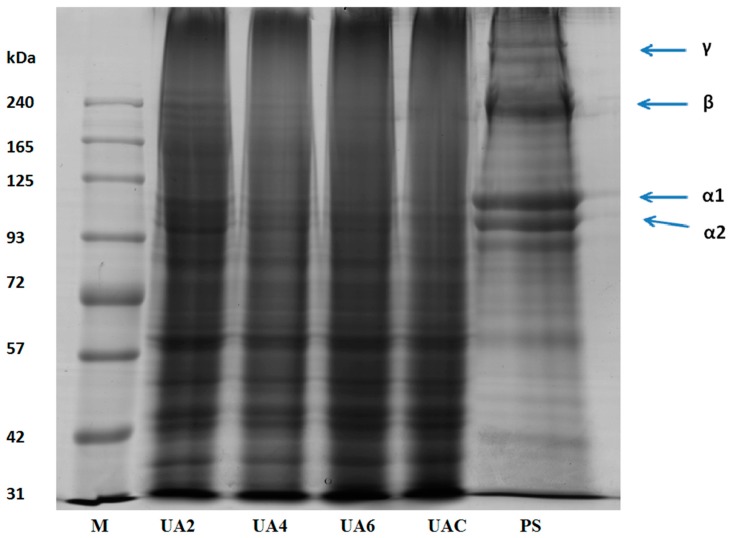
SDS-PAGE pattern of pretreated hide (PS) sample along with gelatin extracted using ultrasound for the time duration of 2, 4 and 6 h (UA2, UA4 and UA6, respectively) from bovine hide with actinidin pretreatment. UAC: control gelatin extracted using ultrasound without enzyme pretreatment. M denotes the marker.

Functional properties of gelatin are affected by the amino acid composition, the molecular weights distribution, structure and compositions of its subunits [34]. Pretreated hide samples (PS), UAC, UA2, UA4, and UA6 samples were subjected to SDS-PAGE analysis (Figure 1). Presence of α1 and α2 chains, β chains (covalently linked α-chains dimers) and γ chains (covalently linked α-chains trimers) were observed in the molecular distribution pattern of pretreated hide samples with highest intensity. UA2 sample revealed the presence of β chains, α1 and α2 chains. Progressive degradation to these chains was observed as the time duration of ultrasound treatment increased. Subsequently, there was complete absence of β and α2 chains in UA6 and very faint presence of β and α2 in UAC. α1 chain was observed in both UAC and UA6. The result showed that the ultrasonic treatment for long duration was responsible for the breakdown of the polypeptides chains. Similar molecular weight distribution pattern was observed for all replicates.

Utilization of ultrasonic in various food products was reported previously. There were no differences in the protein fraction of various food products when ultrasonic treatment was applied for very short durations (i.e., minutes) [50,51,52,53,54,55]. However, there was a decrease in molecular weight when ultrasound treatment of 20 and 40 kHz was applied for 30 min in whey protein concentrate (WPC) and whey protein isolate (WPI) [56] and α-lactalbumin [57]. Degradation of α chains was observed with long duration of ultrasound assisted extraction of gelatin from bighead carp scales [15]. Degradation of protein molecular structure might be due to higher shear stress and turbulence effects of ultrasound treatment [55].

### 2.6. Turbidity

The higher turbidity of UA2 reflected its low quality compared to other samples [22,58]. UA4 and UA6 had significantly (*p* < 0.05) lower turbidity than the UAC (Table 1). This might be due to size reduction of the suspended insoluble aggregates by ultrasound [56]. No earlier reports could be found to compare our results.

### 2.7. Gel Strength

The most significant functional property of gelatin is gel strength which is function of complex interaction decided by molecular weight distribution [22]. Complicated interactions occurring between among amino acid composition and α chain ratio and quantity of β components control the gel strength [34].

The gel strength values of all the ultrasound extracted gelatin (UG) samples were high (Table 1). The highest gel strength value of 627.5 g was found for UAC. The corresponding values for UA2, UA4 and UA6 were 451.5, 520.3 and 502.2 g, respectively. The UA2 sample revealed the presence of β chains along with α1 and α2 chains which degraded progressively and very faint presence of β chain was found in UAC together with only α1 chain. Normally, high molecular weight polypeptides gelatins show high gel strength than the gelatin having low molecular weight distribution [18] because lower weight peptides could not be able to establish inter-junction zones efficiently, failing to form the gelatin chains aggregates leading to low gelling property.

The presence of cross linked two α-chains and the β-component facilitate the peptide chains to regain the triple helical structure when cooled and thereby aids in increasing coiled helix formation during gel maturation resulting in high gel strength [34]. However, the molecular weight distribution and the gelatin molecules aggregate formation could also contribute to the differences in gel strength [34]. Polypeptide chains configuration and the inter-junction zones formed during the maturation process also determine the gel strength [30].

In addition, amino acid composition and the type of extraction treatments also influence the gel strength of gelatin [34]. The imino acid (proline and hydroxyproline) content also governs the gelatin gelling property [59]. Among the two, hydroxyproline is considered the major determining factor for the stability due to its hydrogen bonding ability through the -OH groups [60]. More stable gel structures are formed by the formation of hydrogen bonds by imino acid leading to high gel strength. Significantly (*p* < 0.05) low gel strength of UA2, UA4 and UA6 compared to UAC could be explained by the low proline and hydroxyproline (imino acid) content in these samples, which could be resulted in less organized triple helix structure. Triple helices are partially recovered during maturation of gel and the stability to triple helices is provided by the regions rich in Gly-Pro-Hyp [60].

### 2.8. Viscosity

Viscosity is the second most important commercial physical property of gelatin [60]. Gelatin having high viscosity is commercially valuable [21]. Collagen kept in hot water gets denatured by the breakdown of the hydrogen and probably electrostatic bonds and thus destroying the triple helical structure of collagen to produce one, two or three random chain gelatin molecules that results in a solution in water of high viscosity [21]. Viscosity is partially governed by molecular weight and polydipersity of the gelatin polypeptides [61] meaning that presence of higher molecular weight components increases viscosity but polydispersity can have variable effect depending on the molecular weight distribution [62].

In this study, viscosity values were 16.33, 15.67, 15.87 and 15.60 mPa.s for UAC, UA2, UA4 and UA6, respectively (Table 1). Presence of enzyme decreased the viscosity significantly (*p* <0.05). Viscosity of the commercial bovine gelatin was 9.80 cP [38]. Comparatively high viscosities obtained for these samples might be due to particle sized denatured collagen recovered during ultrasonic extraction attributed to cavitation which caused impingement by micro-jets that resulted in surface peeling, erosion and particle breakdown [11].

### 2.9. FTIR Spectra

Functional groups and secondary structure of gelatin are generally studied using FTIR spectroscopy and the amide I band occurring between 1600 and 1700 cm^−1^ wavenumber is the most crucial to analyse proteins secondary structure using infrared spectroscopy [63]. Amide-I denotes C=O stretching vibration hydrogen bonding coupled with COO, coupled to contributions from the CN stretch, CCN deformation and in-plane NH bending mode [64]. Hydrogen bonding and the conformation of protein structure determines its exact location [65]. Absorption peak at 1633 cm^−1^ is the characteristic of the coiled structure of gelatin [66] and this is in the agreement with our observation of the amide-I peak in the range of 1631–1635 cm^−1^.

**Figure 2:**
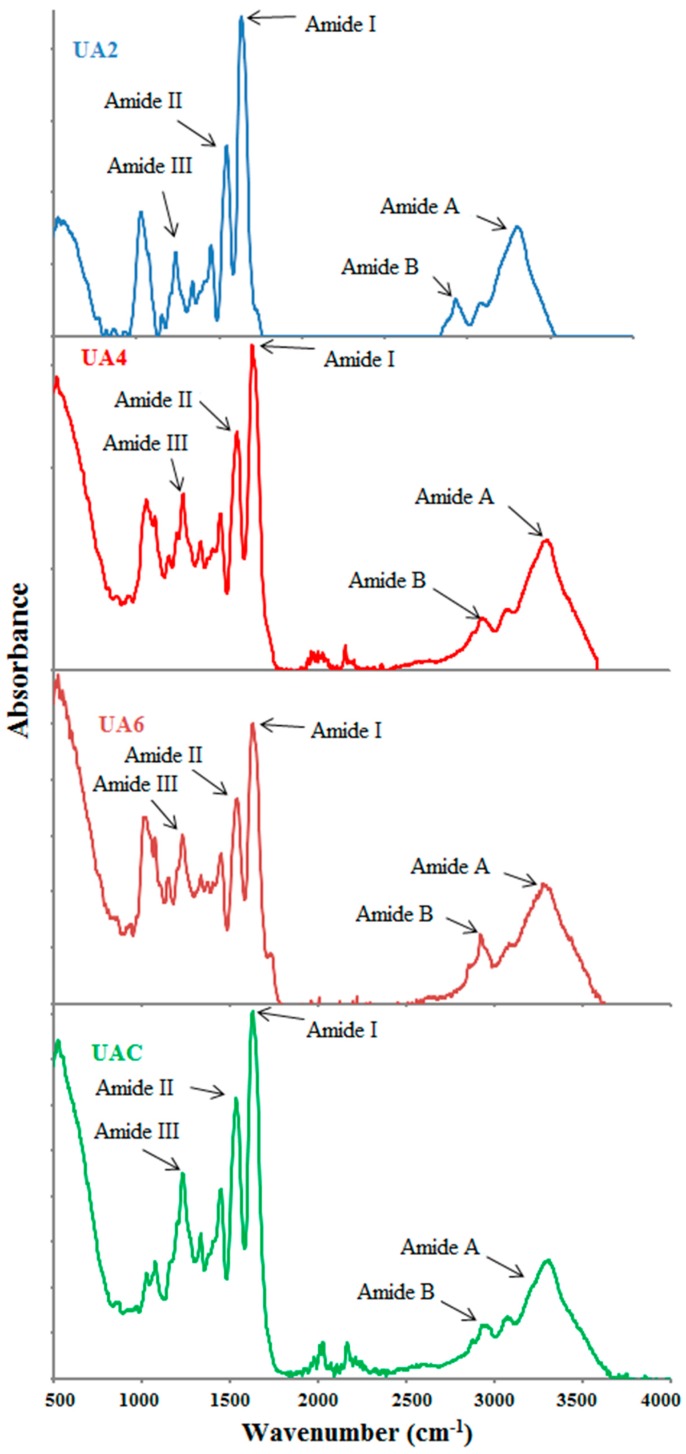
FTIR spectra of gelatin extracted using ultrasound for the time duration of 2, 4 and 6 h (UA2, UA4 and UA6, respectively) with actinidin pretreatment. UAC: control gelatin extracted using ultrasound without enzyme pretreatment.

FTIR spectra of UA2, UA4, UA6 and UAC have been depicted in Figure 2 and peak position of different bands has been presented in Table 4. With slight differences, the major peaks were detected in the amide regions. These spectra were in accordance with those reported by [63]. Amide I bands for UA2, UA4, UA6 and UAC were observed at the wavenumbers of 1632, 1632, 1636 and 1632 cm^−1^, respectively. The amide A amplitudes for all the samples were high and similar. The higher wavenumber along with high amplitude of UA6 showed that the inter-molecular crosslinks were opened thermally resulting in higher loss of molecular order [30] indicating that longer duration of ultrasound treatment along with actinidin pretreatment had caused increased thermal uncoupling of inter-molecular crosslink. Tu et al. [15] also reported higher amide I band for gelatin extracted by ultrasound treatment than that extracted by waterbath method.

Amide II also show alteration in the gelatin secondary structure [67] but specifically it reflects more about the degree of gelatin hydration than its structure [29]. Amide II vibrational modes indicate an out-of-phase combination of CN stretch and in-plane NH deformation modes of the peptide group [30]. Dry collagen had the amide II band in the infrared spectrum range of 1530–1540 cm^−1^ and often had minor bands at lower frequencies [15]. The shifting of amide II to lower wavenumber with lower amplitude suggested H-bond formation with adjacent chains by NH groups [60]. The characteristic absorption bands of UA2, UA4, UA6 and UAC gelatin in amide-II region were shifted to lower wavenumber as the time duration for ultrasound irradiation increased and were observed at the wavenumbers of 1547, 1543, 1539 and 1539 cm^−1^, respectively indicative of higher NH group involvement in hydrogen bonding particularly in UA6 since its showed lower wavenumber and amplitude. Although the amplitudes of UB2, UA4 and UA6 were lower compared to UAC, UA2 and UA4 displayed amide II at higher wavenumber than UAC. 

Amide III spectra for UA2, UA4, UA6 and UAC gelatin were detected at wavenumber of 1238, 1238, 1242 and 1234 cm^−1^. Amide III absorption spectra represents a complex vibrational mode having components due to C-N stretching and N-H in plane bending arising due to amide linkages as well as significant absorptions arising from the wagging vibrations of CH_2_ groups from the glycine back bone and proline side chains and this is generally seen in the region of 1200–1400 cm^−1^ [68]. Amide III band displayed in the range of 1233–1234 cm^−1^ suggested triple helical structure loss resulting from disordered gelatin molecules [37]. In addition, lower amplitude exhibited by amide III indicated loss of triple helix structure into random coiled structure resulting from denaturation of collagen into gelatin due to disruption in natural α helix structure of protein chains [63]. The occurrence of amide III of UAC near the 1234 cm^−1^ and its relatively lower amplitude compared to other treatment samples indicated loss of triple helical structure in UAC. Additionally, some more peaks for all the samples were observed at lower than amide III regions because of stretching vibrations of C-O group present in the smaller peptides [68].

Amide A band arising from NH-stretching coupled with hydrogen which is detected in the range of wavenumber of 3400–3440 cm^−1^ for gelatin samples [63] and involvement of N-H group of a peptide in hydrogen bonding shifts this band to lower wavenumber of around 3300 cm^−1^ [15]. The amide A band of the triple-helix biopolymer shifted to lower frequencies because of hydrogen bond formation by the N-H group of a peptide [28]. For samples UA2, UA4, UA6 and UAC, amide A appeared at 3302, 3310, 3279 and 3291 cm^−1^, respectively. The lower wavenumber of UA6 and UAC compared to UA2 and UA4 indicated higher hydrogen bond formation with the participation of N-H group in α chains. Shifting to lower wavenumber as well as high amplitude of amide A suggested gelatin degradation [28]. The lowest wavenumber along with high amplitude of UA6 implied degraded gelatin. Although UAC displayed amide A at lower wavenumber but its amplitude was lowest amongst the samples. The concurrent effect of actinidin and ultrasound might have brought this difference between the UA6 and UAC.

Asymmetric stretching vibration of =C-H and NH_3_^+^ is represented by amide B bands [30]. The amide B for UA2, UA4, UA6 and UAC were discovered at 2928, 2924, 2936 and 2936 cm^−1^, respectively. Lower wavenumber of UA2 and UA4 compared to UA6 and UAC suggested higher interaction of -NH_3_ group between peptide chains in UA2 and UA4 [28,30]. Thus, it can be concluded that the secondary structures and functional groups were affected by the ultrasound duration and enzyme pretreatment.

### 2.10. Microstructure of Gelatin

**Figure 3:**
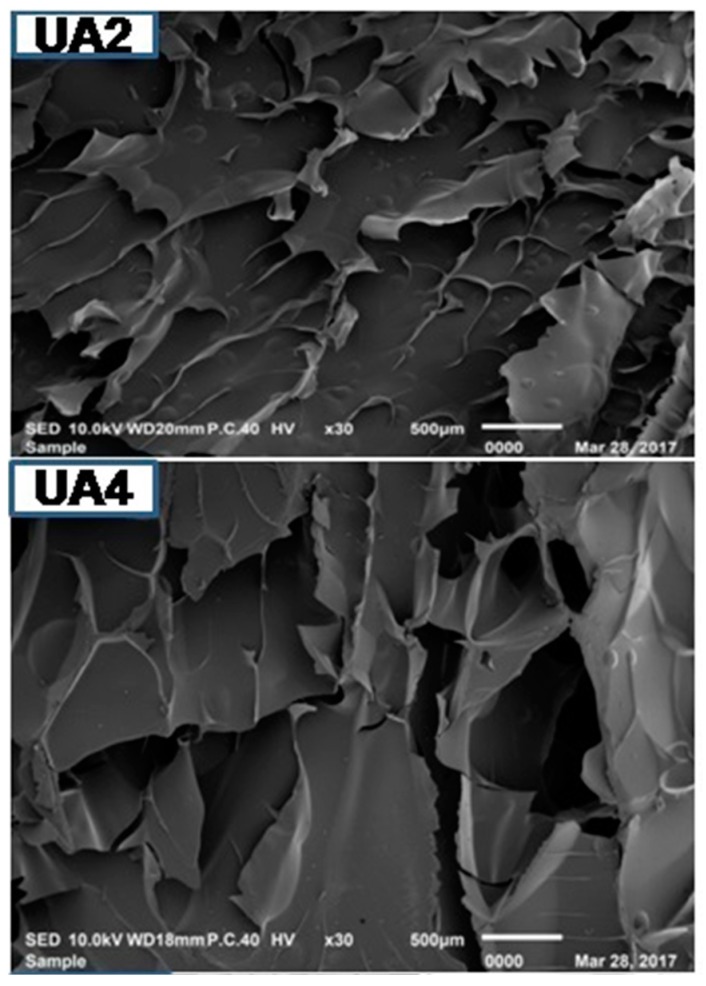

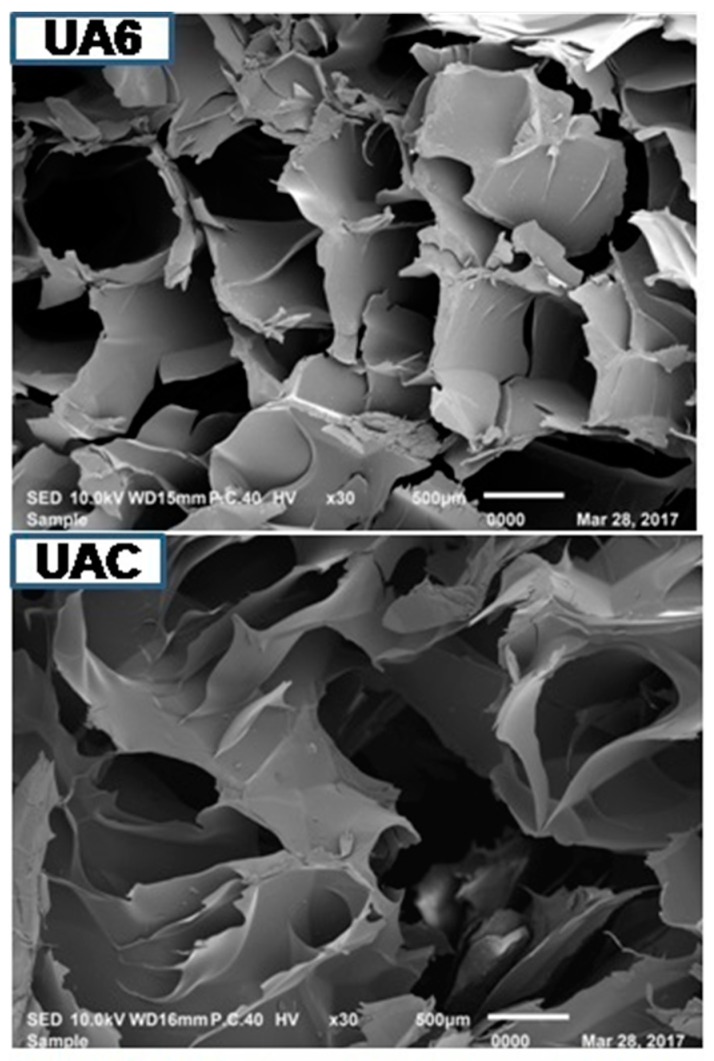
SEM images of gelatin extracted using ultrasound for the time duration of 2, 4 and 6 h (UA2, UA4 and UA6, respectively) with actinidin pretreatment. UAC: control gelatin extracted using ultrasound without enzyme pretreatment.

Microstructure of gelatin is associated with the gelatin physical properties. UA2 displayed less dense, sheet-like structure having particles of bigger size compared to other samples. As the duration of ultrasonic treatment increased, the gelatins structure became denser, inter-connected and disorganized with increasing smaller particles size with increasing voids (Figure 3). Density of the structure increased with the duration of ultrasonic treatment. The particles size of UA6 was smaller and more disorganized than UAC. This might be due to proteolytic degradation by actinidin. Partial unfolding of protein took place under high-power ultrasound whereby, functional groups (such as hydrophobic groups) were exposed and this led to immediate interaction with each other resulting in protein aggregation and network formation [69]. Taking into account the gel strength of the different samples, it seemed that rather than voids, it was the density, large particles size and absence of sheet structure that had more assertive positive effects on the physical properties of gelatin. The result indicated that actinidin pretreatment with ultrasonication resulted in change in the gelatin microstructure.

## 3. Materials and Methods

### 3.1. Chemicals

Acrylamide, sodium dodecyl sulphate (SDS), *N*,*N*,*N*′,*N*′-tetramethyl ethylene diamine (TEMED), coomassie brilliant blue R-250, 2-mercaptoethanolwere purchased from Merck, Darmstadt, Germany. Other chemicals and reagents used were of analytical grade. Enzyme actinidin (>30 casein unit/g) obtained from kiwi fruit (*Actinidadeliciosa*) was kindly gifted by Ingredient Resources Pty Ltd., Warriewood, NSW, Australia. Reagents and amino acid standards were purchased from Waters Corporation, Milford, MA, USA and hydroxyproline standard supplement was procured from Agilent Technologies, Santa Clara, CA, USA. The internal standard (*S*)-(+)-2-Aminobutyric Acid (AABA) was purchased from TCI (Tokyo Chemicals Industry Co., Ltd., Chuo-ku, Japan).

### 3.2. Preparation of Hide

Hide from three- to four-year-old female Brahma cross was procured from a local commercial ruminant abattoir located in Shah Alam, Selangor, Malaysia and transported in ice and stored at −20 °C. The subcutaneous fat was removed by scrapping. The hide was washed thoroughly and stored at −20 °C until further gelatin extraction. It was thawed overnight at 4 °C before being used.

### 3.3. Ultrasound Assisted Extraction of Gelatin from Bovine Hide in Conjugation with Enzyme Actinidin

#### 3.3.1. Removal of Non-Collagenous Proteins

The non-collagenous materials were removed by treating the hide with 0.1 M NaOH (*w*/*v*) solution at a hide/solution ratio of 1:5 (*w*/*v*) stirred at room temperature (25 ± 1 °C) for 6 h, and solution was changed at every 2 h interval. Thereafter, the hairs on the hide were removed by scrapping with scalpel and cut into 1 cm × 2 cm size. The hide was rinsed thoroughly with distilled water until neutral pH wash water was obtained.

#### 3.3.2. Ultrasound Assisted Gelatin Extraction in Conjugation with Enzyme Actinidin

The hide was soaked in 1% HCl for 20 h with discontinuous stirring at a ratio of 1:10 (*w*/*v*) at room temperature for swelling. The samples were washed thoroughly with distilled water until neutral wash water was obtained. From our previous unpublished results, the level of 20 unit/g of actinidin was found to improve the extraction and quality characteristics of gelatin. Therefore, the swollen hides were incubated with enzymes actinidin for 48 h at the level of 20 unit per g of wet hide at their optimum temperature and pH (20 °C and 7.5, respectively) as indicated by the manufacturers.

The swollen hide samples were kept in the optimum pH solution at hide to solution ratio of 1:3 (*w*/*v*) and the enzymes were added. The mixture was kept in the orbital shaker incubator (LM-510RD, Yihder, Xinbei, Taiwan) at 20 °C and stirred for 48 h. Thereafter, the mixture was kept in water bath at 90 °C for 15 min to terminate the enzyme activity. Gelatin was extracted at 60 °C for the time duration of 2, 4 or 6 h in ultrasonic bath (SK8210HP, Kudos, Shanghai, China) using 53 kHz frequency and ultrasonic power of 500 W. The mixture was filtered using cheese cloth and then centrifuged (Beckman Coulter Avanti J-26 XPI, Brea, CA, USA) at 12,800× *g* for 20 min. The supernatant was dried using freeze drier (Labconco FreeZone^18^, Kansas City, MO, USA) and the dry matter obtained, referred to as “gelatin powder”, was stored at 4 °C for further analysis. Control gelatin was extracted using ultrasound treatment at 60 °C for 6 h without the above mentioned enzymatic treatment. The extraction was performed in triplicate.

### 3.4. Analyses of Gelatin

#### 3.4.1. Yield

The yield of the gelatin was calculated on the wet weight basis of the hide as reported previously [34,35,36,70].

$$\mathrm{Yield}\;\left(\%\right)=\frac{\mathrm{Weight}\;\mathrm{of}\;\mathrm{the}\;\mathrm{freeze}\;\mathrm{dried}\;\mathrm{gelatin}\;\left(g\right)\;\times \;100}{\mathrm{Wet}\;\mathrm{weight}\;\mathrm{of}\;\mathrm{the}\;\mathrm{hide}\;\left(g\right)}$$

#### 3.4.2. Determination of Colour

ColorFlexHunterLab (Hunter Associates Laboratory Inc., Reston, VA, USA) was used to measure the colour of the gelatin samples. Three colour co-ordinates, namely L* (lightness), a* (redness/greenness) and b* (yellowness/blueness) were used [71]. The sample was filled in a 64 mm glass sample cup with three readings in the same place and triplicate determinations were taken per sample.

#### 3.4.3. Determination of pH

The BSI 757 of British Standard Institute method was used [72]. One percent (*w*/*v*) of gelatin solution (0.2 g in 20 mL distilled water) was prepared and it was cooled to room temperature of about 25 °C. The pH meter (Mettler Toledo, AG 8603, Schwerzenbach, Switzerland) was standardized with pH 4.0 and 7.0 buffers and pH determination was carried out in triplicates.

#### 3.4.4. Determination of Amino Acid Composition

Procedure of Awad et al. [73] was used with a slight modification to determine the amino acid (AA) content of the gelatin samples using high performance liquid chromatography (HPLC, Milford, MA, USA).Shortly, 5 mL of 6 M HCl was used to hydrolyse 0.1 to 0.2 g of sample at 110 °C for 24 h. Upon cooling, 4 mL of internal standard (l-amino-*N*-butyric acid; AABA) was added to the hydrolysate and aliquot was paper and syringe filtered. Ten microlitres of the filtered sample was mixed with 70 µL of borate buffer and 20 µL of ACCQ reagent (Waters Corporation, Milford, MA, USA). A mixture of amino acid standard H (Waters Corporation, MA, USA) and the AABA internal standard (TCI, Chuo-ku, Japan) was spiked with hydroxyproline (Agilent Technologies, CA, USA). The resulting solution was used for derivatization as the working standard. The method was followed to determine the concentration of all AA except methionine, cysteine and tryptophan. Then, an AA column (AccQ Tag 3.9 150 mm; Waters Corporation, MA, USA) was used for peaks separation. Peaks were detected by a fluorescent detector (2475; Waters Corporation, MA, USA). Triplicate determinations were performed and data corresponds to mean values. Standard deviations in all cases lower than 2%.

#### 3.4.5. Electrophoretic Analysis

The molecular weight distributions of the extracted gelatins were determined by SDS-PAGE [74]. Dry gelatin (10 mg) was dissolved in distilled water (1 mL) at 60 °C to create a 10 mg/mL solution. The sample solution was mixed in a 1:2 (*v*/*v*) ratio with a 5-fold-concentrated loading buffer (3.55 mL deionized water, 1.25 mL 0.5 M Tris-HCl, pH 6.8, 2.5 mL glycerol, 2.0 mL 10% (*w*/*v*) SDS, 0.2 mL 0.5% (*w*/*v*) bromophenol blue) containing β-mercaptoethanol (50 µL β-mercaptoethanol+ 950 µL sample buffer prior to use). The mixed solution was heated in boiling water (95 °C) for 5 min before loading onto 4% stacking gels and 7.5% resolving gels. Gel electrophoresis (Mini-PROTEAN Tetra System, Bio-Rad Laboratories, Irvine, CA, USA) was run at a constant current of 15 mA/gel for 15 min; followed by 25 mA/gel until the bromophenol blue dye reached at the bottom of the gel. Following electrophoresis, the gel was stained with 0.1% (*w*/*v*) coomassie blue R-250 in 15% (*v*/*v*) methanol and 5% (*v*/*v*) acetic acid for 2 h and destained with 30% (*v*/*v*) methanol and 10% (*v*/*v*) acetic acid until the zones on the blue background were clear. Prestained protein ladder (BLUeye, GeneDireX, Keelung City, Taiwan) was used to estimate the molecular weight distributions of the gelatins. The gel was scanned with a GS-800 Calibrated Densitometer (Bio-Rad Laboratories, CA, USA) gel imaging system.

#### 3.4.6. Determination of Turbidity

Method of Cho et al. [75] was modified slightly to determine the turbidity of the gelatin solutions. Gelatin sample (0.025 g) was dissolved in distilled water (5 mL) at 60 °C to make 0.5% (*w*/*w*) solution. Absorbance was measured at 660 nm by spectrophotometer (Shimadzu UV Spectrophotometer, Model UV-1800, Kyoto, Japan).

#### 3.4.7. Determination of Gel Strength

Method of Fernandez-Dıaz [76] was slightly modified to determine the gel strength of the extracted gelatin. Gelatin (2.0 g) was dissolved in 30 mL of distilled water at 60 °C using 50 mL-beaker (Schott Duran, Mainz, Germany) to get the final concentration of 6.67% (*w*/*v*). The solution was stirred until gelatin was solubilized completely, and kept at 7 °C for 16–18 h for gel maturation. Bloom strength was measured using Model TA-XT2*i* Texture Analyzer (Stable Micro Systems, Surrey, UK) using a load cell of 5 kN equipped with a 1.27 cm diameter flat-faced cylindrical Teflon plunger (P/0.5R). The dimensions of the sample were3.8 cm in diameter and 2.7 cm in height. The maximum force (in grams) was recorded when the probe penetrated a distance of 4mm inside the sample. The speed of the plunger was 0.5 mm/s. All determinations are means of three measurements.

#### 3.4.8. Determination of Viscosity

Gelatin solution of 6.67% was prepared by dissolving 1.34 g of gelatin in 20 mL of distilled water and heated to 60 °C. RheolabQC (Anton Paar, Graz, Austria) viscometer was used to measure the viscosity of the samples. The measurement was performed in triplicate.

#### 3.4.9. Fourier Transform Infrared (FTIR) Spectroscopy 

FTIR spectra were obtained using spectrometer (Perkin Elmer Ltd., Model: Spectrum 100, Tempe, AZ, USA) equipped with a deuterated triglycine sulphate (DTGS) detector. The attenuated total reflectance (ATR) accessory was mounted into the sample compartment. Diamond internal reflection crystal had a 45° angle of incidence to the IR beam. Resolution of 4 cm^−1^ was used to acquire the spectra and 4000–500 cm^−1^ (mid-IR region) was chosen as measurement range at room temperature. Automatic signals were collected in 16 scans and were normalized against a background spectrum recorded from the clean, empty cell at 25 °C.

#### 3.4.10. Microstructure Analysis of Gelatin

Scanning electron microscope (SEM) (JEOL JSM-IT100 InTouchScope, Tokyo, Japan) was used to elucidate the microstructures of gelatin. Dried gelatin samples having a thickness of 2–3 mm were mounted on a bronze stub and sputter-coated with gold (BAL-TEC SCD 005 sputter coater, Schalksmühle, Germany). An acceleration voltage of 10 kV was used to observe the specimen at 30×.

### 3.5. Statistical Analysis

All statistical analyses were carried out using GLM procedure of Statistical Analysis System package (SAS) Version 9.4 software (Statistical Analysis System, SAS Institute Inc., Cary, NC, USA) and statistical significance was set at *p* < 0.05. Significant differences between means were evaluated by Duncan’s Multiple Range Test.

## 4. Conclusions

Ultrasonication (53 kHz and 500 W) for 6 h at 60 °C can significantly increase the gelatin recovery in conjugation with enzyme actinidin pretreatment. The obtained gelatin showed higher gel strength and viscosity. SDS-PAGE analysis showed progressive degradation of protein chains as the time duration of ultrasound treatment increased. UA2 samples revealed the presence of β, α1 and α2 chains but there was complete absence of β and α2 chains in UA6 and very faint presence of β and α2 chains in UAC. Both UAC and UA6 showed the presence of α1 chain. Amino acids content of the extracted gelatin increased as the time duration of ultrasonic treatment increased. FTIR spectra demonstrated greater loss of molecular order in UA6 and its degradation which might be due to thermal uncoupling of inter-molecular crosslink resulting from longer duration of ultrasound treatment and actinidin pretreatment. SEM images indicated increasing time of ultrasound extraction caused protein aggregation and network formation in the gelatins resulting in increased density and decreased structural integrity.

## Figures and Tables

**Table 1:** Yields, pH, turbidity, gel strength and viscosity of gelatin extracted using ultrasound from bovine hide pretreated with enzyme actinidin. Values are presented as mean ± SE from triplicate determination.

Sample	Yield (%) of Gelatin	pH	Turbidity (ppm)	Gel Strength (g)	Viscosity (mPa.s)
UAC	18.72 ± 018 ^b^	2.91 ± 0.02 ^c^	53.28 ± 0.47 ^b^	627.5 ± 4.48 ^a^	16.33 ± 0.03 ^a^
UA2	8.64 ± 0.08 ^d^	2.75 ± 0.01 ^d^	105.53 ± 0.15 ^a^	451.5 ± 5.29 ^d^	15.67 ± 0.03 ^c^
UA4	15.17 ± 0.18 ^c^	2.97 ± 0.01 ^b^	25.98 ± 0.27 ^d^	520.3 ± 4.18 ^b^	15.87 ± 0.06 ^b^
UA6	19.65 ± 0.19 ^a^	3.03 ± 0.02 ^a^	32.03 ± 0.15 ^c^	502.2 ± 4.06 ^c^	15.60 ± 0.04 ^c^

^a, b, c, d^ Means with different superscripts in the same column indicate significant difference at *p* < 0.05. UA2, UA4 and UA6 refers to ultrasound assisted gelatin extracted for the time duration of 2, 4 and 6 h, respectively using actinidin pretreatment. UAC: control gelatin extracted using ultrasound without enzymatic pretreatment.

**Table 2:** Colour of gelatin extracted using ultrasound from bovine hide pretreated with enzyme actinidin. Values are presented as mean ± SE from triplicate determination.

Treatment	L*	a*	b*
UAC	64.45 ± 0.29 ^b^	1.91 ± 0.02 ^b^	17.10 ± 0.20 ^a^
UA2	73.15 ± 0.23 ^a^	0.26 ± 0.05 ^c^	10.27 ± 0.18 ^d^
UA4	62.64 ± 0.09 ^c^	2.47 ± 0.05 ^a^	16.40 ± 0.21 ^b^
UA6	63.43 ± 0.55b ^c^	1.84 ± 0.07 ^b^	14.57 ± 0.23 ^c^

^a, b, c, d^ Means with different superscripts in the same column indicate significant difference at *p* < 0.05. UA2, UA4 and UA6 refers to ultrasound assisted gelatin extracted for the time duration of 2, 4 and 6 h, respectively using actinidin pretreatment. UAC: control gelatin extracted using ultrasound without enzymatic pretreatment.

**Table 3:** Amino acid composition (per centof gelatin sample) of gelatin samples. UA2, UA4 and UA6 refers to gelatin extracted using ultrasound for the time duration of 2, 4 and 6 h, respectively from bovine hide pretreated with enzyme actinidin. UAC: control gelatin extracted using ultrasound without enzymatic pretreatment.

Amino Acids	Gelatin Samples			
	UAC	UA2	UA4	UA6
Hydroxyproline (Hyp)	17.00	10.77	12.64	13.65
Aspartic acid (Asp)	2.99	3.02	3.29	3.28
Serine (Ser)	3.30	2.30	2.64	2.91
Glutamic acid (Glu)	8.28	6.07	6.61	6.96
Glycine (Gly)	25.54	16.86	18.95	20.60
Histidine (His)	0.96	0.63	0.71	0.74
Arginine (Arg)	8.41	5.43	6.31	6.89
Threonine (Thr)	1.91	1.29	1.49	1.58
Alanine (Ala)	7.64	5.43	6.06	6.31
Proline (Pro)	11.39	8.33	9.26	9.78
Tyrosine (Tyr)	0.66	0.40	0.46	0.50
Valine (Val)	2.18	1.60	1.78	1.87
Lysine (Lys)	3.28	2.37	2.72	2.79
Isoleucine (Ile)	1.34	0.95	1.07	1.12
Leucine (Leu)	2.81	2.02	2.26	2.35
Phenylalanine (Phe)	1.99	1.37	1.55	1.64
Imino acids (Pro + Hyp)	28.39	19.10	21.90	23.43

**Table 4:** FTIR spectra peak position of gelatin samples extracted for the time duration of 2, 4 and 6 h (UA2, UA4 and UA6, respectively) using ultrasound in conjugation with actinidin pretreatment at level 20 units/g of hide. UAC: control gelatin extracted using ultrasound (U) without enzyme pretreatment.

Band	Peak Wavenumber (cm^−1^)			
	UA2	UA4	UA6	UAC
Amide I	1632	1632	1636	1632
Amide II	1547	1543	1539	1539
Amide III	1238	1238	1242	1234
Amide A	3302	3310	3279	3291
Amide B	2928	2924	2936	2936
